# Incidence and Prognostic Significance of PD-L1 Expression in High-Grade Salivary Gland Carcinoma

**DOI:** 10.3389/fonc.2021.701181

**Published:** 2021-08-26

**Authors:** Qigen Fang, Yao Wu, Wei Du, Xu Zhang, Defeng Chen

**Affiliations:** Department of Head Neck and Thyroid, Affiliated Cancer Hospital of Zhengzhou University, Henan Cancer Hospital, Zhengzhou, China

**Keywords:** salivary gland carcinoma, PD-L1, immunotherapy, high-grade salivary gland carcinoma, survival

## Abstract

**Objective:**

PD-L1 is one of the predictors of immunotherapy efficacy. Our goal was to analyze its expression and prognostic significance in high-grade salivary gland carcinoma (SGC).

**Methods:**

PD-L1 expression was evaluated using paraffin-embedded specimens from patients with surgically treated high-grade SGC, and it was scored by the tumor proportion score (TPS), combined positive score (CPS), and immune cell (IC) score. Associations between clinicopathological variables, disease-free survival (DFS), overall survival (OS) and PD-L1 expression were assessed.

**Results:**

TPS≥1% occurred in 47 patients with an incidence of 43.1%, and it was significantly related to an advanced tumor stage. In patients with TPS<1%, TPS ranging from 1% to 20%, and TPS≥20%, the 5-year DFS rates were 36%, 26%, and 13%, respectively, and the difference was significant. In patients with TPS<1%, TPS ranging from 1% to 20%, and TPS≥20%, the 5-year OS rates were 49%, 24%, and 13%, respectively, and the difference was significant. CPS≥1 occurred in 87 patients with an incidence of 79.8%. IC scores of 0, 1, 2, and 3 were noted in 24 (22.0%), 37 (33.9%), 31 (28.4%), and 17 (15.6%) patients, respectively. Both CPS and IC scores had no impact on DFS or OS.

**Conclusions:**

The expression of PD-L1 in tumor cells of high-grade SGCs was not uncommon, and it was significantly associated with tumor stage. PD-L1 expression in tumor cells rather than in immune cells indicated a poor prognosis.

## Introduction

Salivary gland carcinoma (SGC) is a relatively uncommon malignancy and accounts for less than 10% of all head and neck cancers (1). Based on the 2017 WHO classification, SGCs consist of 24 different histologic types (2). Due to their different biological behaviors and prognoses, SGCs are divided into three grades: low, intermediate, and high (3). Usually, high-grade SGC is the least frequent but has the worst prognosis. Even when treated with systemic therapies, many patients can still develop a recurrence (4, 5). More effective treatments are required to improve their prognosis.

Immune checkpoint inhibitors, such as programmed death 1 (PD-1) inhibitors, have been confirmed to be effective in controlling many malignant tumors (6). Expression of programmed death ligand-1 (PD-L1) is recognized as an important predictor of immunotherapy efficacy. A number of pioneers have analyzed PD-L1 expression in SGCs (7–9), but conflicting data have been reported, some researchers have described that about 20% of the patients show PD-L1 expression in SGC cells, and it is associated with poor disease free survival and overall survival (7, 8), but some have noted there is little relationship between PD-L1 expression and the disease specific survival (9). The scientific value of these studies is limited by no uniform standards of cutoff values, tissue specimens, antibodies, and scoring criteria for evaluating PD-L1 expression, there is still a lot of unknown knowledge regarding expression pattern and prognostic significance of PD-L1 especially in high grade SGCs, which is rarely analyzed, as far as we know, there are only four reports available for learning (10–13), according to the literature, the incidence of PD-L1 expression ranged from 26% to 53%, Xu et al. (10) and Sato et al. (11) would agree that high PD-L1 expression in salivary duct carcinoma was strongly associated with unfavorable prognosis, but Hamza et al. (12) and Schvartsman et al. (13) might not support this statement but presented PD-L1 expression had no effect on the survival. Therefore, in the current study, we aimed to evaluate the expression pattern and survival significance of PD-L1 in high-grade SGCs to explore the potential benefit of immunotherapy in this specific group.

## Patients and Methods

### Ethics

Our hospital institutional research committee approved this study, and all participants signed an informed consent agreement. All procedures performed were in conducted in accordance with the ethical standards of the institutional and/or national research committee and the 1964 Helsinki Declaration and its later amendments or comparable ethical standards.

### Patient Selection

From January 2010 to January 2021, the medical records of patients with surgically treated SGCs were retrospectively reviewed, and the enrolled patients met the following criteria: the disease was primary and classified as high grade based on the 2017 WHO classification (2); there was no history of other cancers; and there was enough paraffin-embedded tissue available for the PD-L1 expression test. Patients without sufficient demographic, pathologic, or follow-up data were excluded from the analysis. Information regarding age, sex, TNM stage (8^th^ AJCC system), pathologic reports, treatment, and follow-up was extracted and analyzed.

### PD-L1 Expression Test

PD-L1 expression was tested by immunohistochemistry staining using 4μm thick sections of formalin-fixed, paraffin-embedded specimens and a monoclonal antibody targeting PD-L1 (SP263). The antibody was intended for diagnostic use *in vitro* and was employed according to the instructions of the manufacturer’s protocol. The sections were rehydrated through graded ethanol at room temperature followed by deparaffinized in xylene. They were incubated with primary antibody for 30 minutes firstly, and then with biotinylated secondary antibodies. Immunoreactions were visualized using a 3-amino-9-ethylcarbazole as a substrate (Ventana OptiView DAB IHC detection KIT, Ref: 760-700, Mannheim, Germany). Human non-neoplastic tonsillar tissue was used as a positive control for the antibody.

TPS referred to the percentage of viable tumor cells showing partial or complete membrane PD-L1 staining at any intensity; CPS referred to the number of PD-L1 stained cells (tumor cells, lymphocytes, macrophages) divided by the total number of viable tumor cells multiplied by 100; and IC referred to the percentage of tumor area covered by PD-L1+ immune cells (4-tiered score: 0: <1%, 1: 1-5%, 2: 5-10%, 3: >10%). All PD-L1 expression evaluation was performed with high power microscope (×200).

### Treatment Principle

In our cancer center, a definite diagnosis of SGC is usually made based on postoperative pathology. If a high-grade SGC was confirmed, adjuvant radiotherapy and/or chemotherapy were always administered. Neck dissection was performed if there was a cN+ neck. After discharge from the hospital, the patients were routinely followed every 3 months for the first two years and then every 6 to 12 months for the next 3 years. If disease recurrence was suspected, active interference was immediately performed.

### Statistical Analysis

Associations between clinicopathological variables and PD-L1 expression were evaluated by the chi-square test. The Kaplan-Meier method was used to analyze disease-free survival (DFS) and overall survival (OS). The DFS was calculated from the date of surgery to the date of recurrence or the last follow-up visit, and OS was calculated from the date of surgery to the date of death or last follow-up visit. Factors that were significant in univariate analysis were then analyzed in a Cox proportional hazards regression model to study the independent effects on survival. All statistical analyses were performed by SPSS 20.0, and p<0.05 was considered to be significant.

## Results

### Baseline Information of the Patients

A total of 109 patients were included for analysis; there were 60 (55.0%) men and 49 (45.0%) women, and the mean age was 45.6 ± 10.4 years. Primary sites were distributed in the parotid gland in 55 (50.5%) patients, submandibular gland in 27 (24.8%) patients, sublingual gland in 15 (13.8%) patients, and minor gland in 12 (11.0%) patients.

Tumor stages were classified as T1 in 17 (15.6%) patients, T2 in 45 (41.3%) patients, T3 in 35 (32.1%) patients, and T4 in 12 (11.0%) patients. Neck lymph node stages were classified as N0 in 68 (62.4%) patients and N+ in 41 (37.6%) patients. The most common histologic type was high-grade mucoepidermoid carcinoma (MEC), followed by salivary duct carcinoma (SDC), which occurred in 47 (43.1%) and 33 (30.3%) patients, respectively. Adenocarcinoma, not otherwise specified, developed in 15 (13.8%) patients. Squamous cell carcinoma occurred in 8 (7.3%) patients. The least common histologic types were small cell carcinoma, large cell carcinoma, and spindle cell carcinoma, which all developed in 2 (1.8%) patients each. Perineural invasion (PNI) and lymphovascular invasion (LVI) were noted in 37 (33.9%) and 33 (30.3%) patients, respectively. A positive margin occurred in 9 (8.3%) patients.

All patients underwent surgical treatments, 60 (55.0%) patients also underwent neck dissection, and pathologic lymph node metastasis occurred in 45 (75.0%, 45/60) patients. All patients received adjuvant radiotherapy, and 24 (22.0%) patients also underwent adjuvant chemotherapy (**Table 1**).

**Table 1:** Demographic and pathologic information of the patients.

Variables	Number (%)
Age	
≥45	48 (44.0%)
<45	61 (56.0%)
Gender	
Male	60 (55.0%)
Female	49 (45.0%)
Primary site	
Parotid gland	55 (50.5%)
Submandibular gland	27 (24.8%)
Sublingual gland	15 (13.8%)
Minor gland	12 (11.0%)
Histologic type	
High grade mucoepidermoid carcinoma	47 (43.1%)
Salivary duct carcinoma	33 (30.3%)
Adenocarcinoma not otherwise specified	15 (13.8%)
Squamous cell carcinoma	8 (7.3%)
Small cell carcinoma	2 (1.8%)
Large cell carcinoma	2 (1.8%)
Spindle cell carcinoma	2 (1.8%)
Perineural invasion	37 (33.9%)
Lymphovascular invasion	33 (30.3%)
Tumor stage	
T1-T2	62 (56.9%)
T3-T4	47 (43.1%)
Neck lymph node stage	
N0	68 (62.4%)
N+	41 (37.6%)
Positive margin	9 (8.3%)
Radiotherapy	109 (100%)
Chemotherapy	24 (22.0%)

### PD-L1 Expression

TPS≥1% occurred in 47 patients with an incidence of 43.1%; in these 47 patients, 32 cases had a TPS<20%, and 15 cases had a TPS≥20%. CPS≥1 occurred in 87 patients with an incidence of 79.8%; of these 87 patients, 47 had a CPS<20, and 40 had a CPS≥20. IC scores of 0, 1, 2, and 3 were noted in 24 (22.0%), 37 (33.9%), 31 (28.4%), and 17 (15.6%) patients, respectively.

### Predictors of PD-L1 Expression

As **Table 2** describes, in patients with T1-T2 tumors, 42 cases had TPS<1%, 17 cases had a TPS ranging from 1% to 20%, and 3 cases had a TPS≥20%; in patients with T3-T4 tumors, 20 cases had TPS<1%, 15 cases had a TPS ranging from 1% to 20%, and 12 cases had a TPS≥20%, and the difference was significant (p=0.003). No apparent associations between any other variables and TPS were noted (all p>0.05). Furthermore, there were no significant relationships between any of the clinicopathological variables and the CPS or IC score (all p>0.05).

**Table 2:** Association between clinicopathological variables and PD-L1 expression.

Variable	Tumor proportion score			p	Combined positive score			p	Immune cell score		p
	<1%	1-20%	≥20%		<1	1-20	≥20		0/1	2/3	
Age											
≥45	27	16	5		10	23	15		29	19	
<45	35	16	10	0.558	12	24	25	0.557	32	29	0.406
Gender											
Male	33	18	9		13	27	20		35	25	
Female	29	14	6	0.882	9	20	20	0.717	26	23	0.581
Primary site											
Parotid gland	32	16	7		10	26	19		27	28	
Submandibular gland	16	7	4		7	10	10		16	11	
Sublingual gland	8	5	2		3	6	6		9	6	
Minor gland	6	4	2	0.997	2	5	5	0.973	9	3	0.386
Histologic type											
High grade mucoepidermoid carcinoma	28	12	7		9	20	18		26	21	
Salivary duct carcinoma	19	9	5		8	15	10		20	13	
Adenocarcinoma not otherwise specified	7	6	2		3	7	5		8	7	
Others	8	5	1	0.932	2	5	7	0.931	7	7	0.910
Perineural invasion	22	10	7	0.588	8	17	12	0.803	20	17	0.232
Lymphovascular invasion	19	8	6	0.588	6	17	10	0.498	19	14	0.823
Tumor stage											
T1-T2	42	17	3		14	27	21		37	25	
T3-T4	20	15	12	0.003	8	20	19	0.695	24	23	0.370
Neck lymph node stage											
N0	43	16	9		13	30	25		39	29	
N+	19	16	6	0.182	9	17	15	0.931	22	19	0.707

### Survival Analysis

After a follow-up with a mean time of 3.8 (range: 0.6-9.6) years, disease recurrence occurred in 71 patients, and 62 patients died. The overall 5-year DFS and OS rates were 30.0% and 36%, respectively.

**Figure 1:**
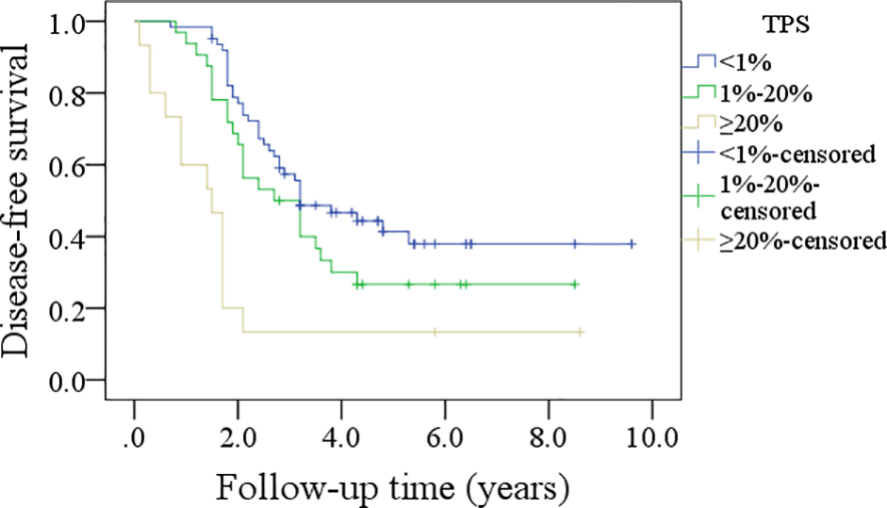
Comparison of disease-free survival in patients with different tumor proportion scores (TPSs), combined positive score (CPS), and immune cell (IC) score.

**Figure 2:**
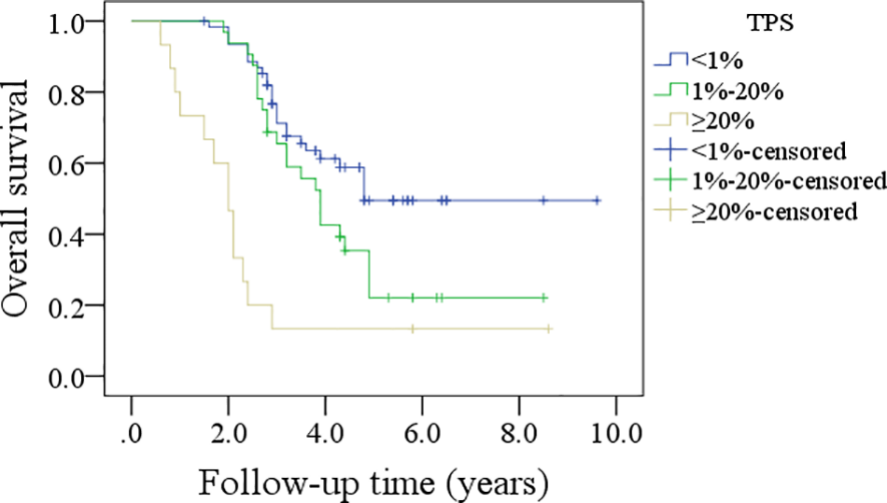
Comparison of overall survival in patients with different tumor proportion scores (TPSs).

In patients with TPS<1%, the 5-year DFS rate was 36%; in patients with TPS ranging from 1% to 20%, the 5-year DFS rate was 26%; and in patients with TPS≥20%, the 5-year DFS rate was 13%, and the difference was significant (p<0.001, **Figure 1**). In patients with TPS<1%, the 5-year OS rate was 49%; in patients with TPS ranging from 1% to 20%, the 5-year OS rate was 24%; and in patients with TPS≥20%, the 5-year OS rate was 13%, and the difference was significant (p<0.001, **Figure 2**). Further, the Cox model confirmed the independence of TPS’s correlation with DFS and OS (**Tables 3** and **4**).

**Table 3:** Survival effect of PD-L1 expression on disease free survival in high grade salivary gland carcinoma.

Variable	Univariate	Cox model	
	p	p	HR [95%CI]
Age (≥45 *vs* <45)	0.354		
Gender (Male *vs* female)	0.267		
Primary site (Parotid *vs* others)	0.004	0.002	1.896 [1.046-4.332]
Histologic type (Mucoepidermoid carcinoma *vs* others)	0.154		
Perineural invasion	<0.001	<0.001	2.006 [1.356-5.337]
Lymphovascular invasion	<0.001	<0.001	1.675 [1.114-3.008]
Tumor stage (T3+T4 *vs* T1+T2)	<0.001	<0.001	4.675 [2.337-9.988]
Neck lymph node stage (N+ *vs* N0)	<0.001	<0.001	3.285 [2.217-7.447]
Tumor proportion score			
<1%			
1% to 20%		0.015	1.994 [1.175-2.887]
≥20%	<0.001	<0.001	3.063 [2.000-6.326]
Combined positive score			
<1			
1 to 20			
≥20	0.635		
Immune cell score (2/3 *vs* 0/1)	0.600		

**Table 4:** Survival effect of PD-L1 expression on overall survival in high grade salivary gland carcinoma.

Variable	Univariate	Cox model	
	p	p	HR [95%CI]
Age (≥45 *vs* <45)	0.632		
Gender (Male *vs* female)	0.227		
Primary site (Parotid *vs* others)	<0.001	0.002	2.976 [1.365-4.338]
Histologic type (Mucoepidermoid carcinoma *vs* others)	0.641		
Perineural invasion	<0.001	<0.001	2.156 [1.227-4.307]
Lymphovascular invasion	<0.001	<0.001	1.998 [1.032-3.098]
Tumor stage (T3+T4 *vs* T1+T2)	<0.001	<0.001	3.876 [1.578-7.447]
Neck lymph node stage (N+ *vs* N0)	<0.001	<0.001	3.002 [1.674-6.885]
Tumor proportion score			
<1%			
1% to 20%		0.021	2.075 [1.275-4.886]
≥20%	<0.001	<0.001	3.328 [1.998-6.356]
Combined positive score			
<1			
1 to 20			
≥20	0.540		
Immune cell score (2/3 *vs* 0/1)	0.422		

**Figure 3:**
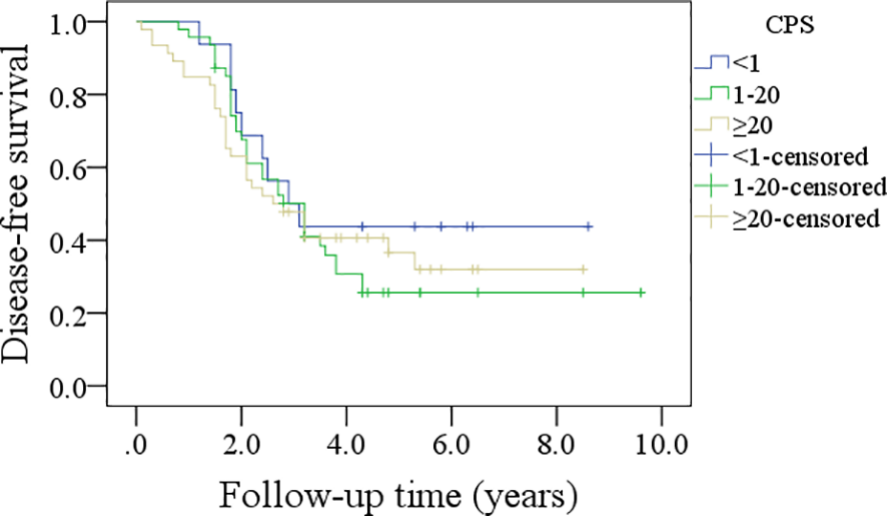
Comparison of disease-free survival in patients with different combined positive scores (CPSs).

**Figure 4:**
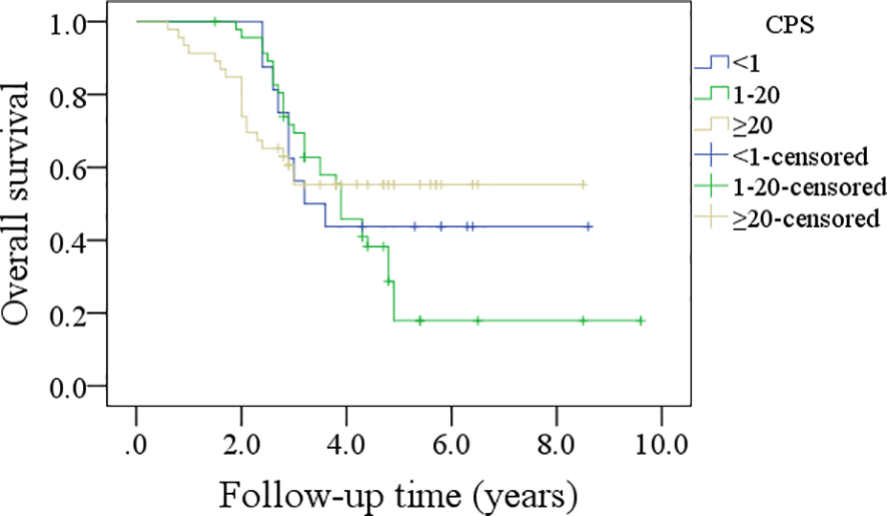
Comparison of overall survival in patients with different combined positive scores (CPSs).

In patients with CPS<1, the 5-year DFS rate was 44%; in patients with CPS ranging from 1 to 20, the 5-year DFS rate was 24%; and in patients with CPS≥20, the 5-year DFS rate was 30%, and the difference was not significant (p=0.635, **Figure 3**). In patients with CPS<1, the 5-year OS rate was 44%; in patients with CPS ranging from 1 to 20, the 5-year OS rate was 21%; and in patients with CPS≥20, the 5-year OS rate was 53%, and the difference was not significant (p=0.540, **Figure 4**).

**Figure 5:**
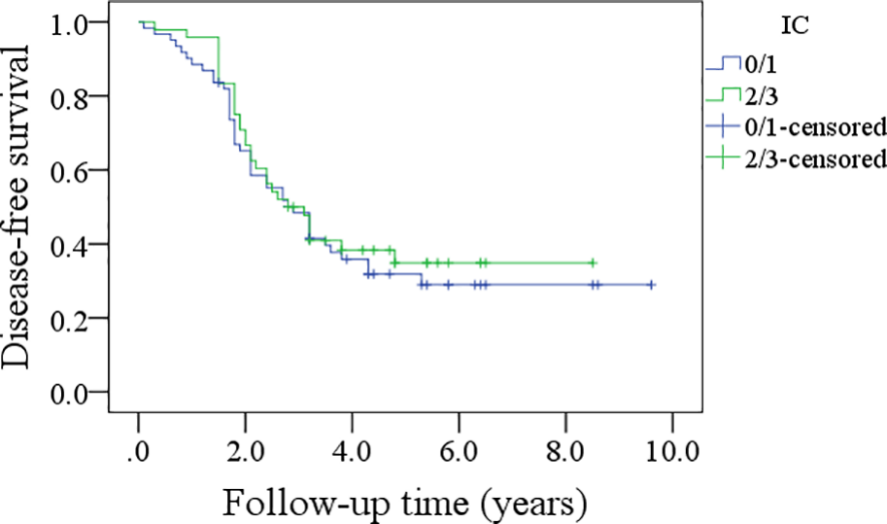
Comparison of disease-free survival in patients with different immune cell (IC) scores.

**Figure 6:**
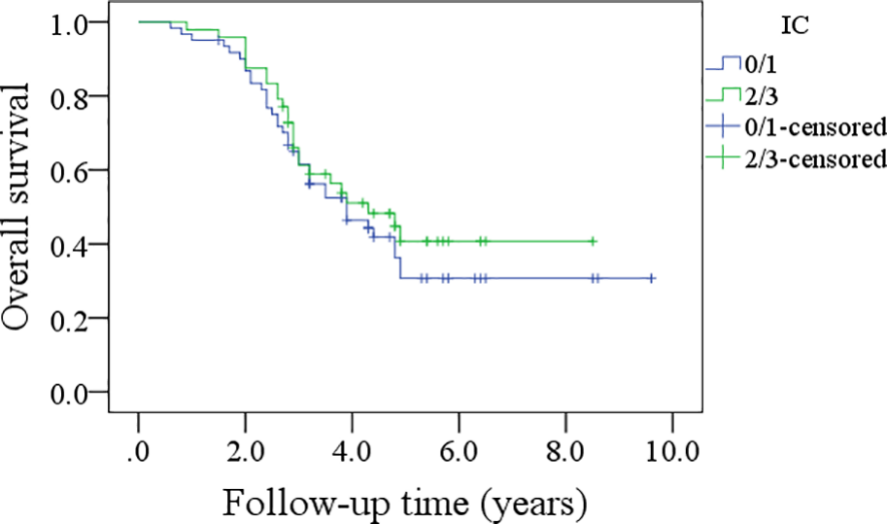
Comparison of overall survival in patients with different immune cell (IC) scores.

In patients with an IC score of 0/1, the 5-year DFS rate was 28%; in patients with an IC score of 2/3, the 5-year DFS rate was 34%, and the difference was not significant (p=0.600, **Figure 5**). In patients with an IC score of 0/1, the 5-year OS rate was 32%; in patients with an IC score of 2/3, the 5-year OS rate was 41%, and the difference was not significant (p=0.422, **Figure 6**).

## Discussion

The most significant finding in the current study was that PD-L1 expression was not uncommon in high-grade SGC cells. TPS was associated with tumor stage and prognosis, and a greater TPS indicated worse survival. Both CPS and IC scores had no relationship with clinicopathological variables or prognosis. Our study provided valuable evidence that TPS could represent a better target for immune checkpoint inhibition than the CPS and IC scores.

The PD-L1/PD-1 axis mediates immune tolerance and promotes tumor growth and progression *via* the inhibition of anti-tumor immunity. Blocking the interaction between PD-L1 and PD-1 was clinically shown to be beneficial in maintaining the anti-tumor functions of the adaptive immune system (14). It was important to explore the PD-L1 expression level in solid malignancies, but it is not frequently analyzed in SGCs. Mukaigawa et al. (7) might be the first to investigate this issue and found that 22.8% of 219 surgically resected SGC specimens had PD-L1 expression. Moreover, the expression of PD-L1 in cancer cells was significantly related to age, sex, tumor location, pathologic tumor and nodal stages, histologic type, and pathologic grade. However, in this study, the authors defined PD-L1 expression positivity as a case showing complete membranous expression of PD-L1 in more than 1% of the carcinoma cells, which was apparently different from other studies. Vital et al. (8) considered positivity for PD-L1 if there was any unequivocal membranous staining of at least 1% of the tumor cells; in their 167 SGC patients, 17% showed PD-L1 positivity, and PD-L1 expression in tumor cells was associated with a higher tumor grade. A similar definition of PD-L1 expression positivity was used by Higashino et al. (9); the authors reported a rate of 28.3%, and it was more common in tumors with a higher stage, a higher grade, and node-positive cases. However, none of these three studies focused on high-grade SGCs, which usually have a worse prognosis than low- and intermediate-grade SGCs and deserve more attention (4, 15).

Very few researchers have examined the significance of PD-L1 expression in high-grade SGCs. Hamza et al. (12) evaluated salivary duct carcinoma specimens from 113 patients and found that 26% of the samples had positive PD-L1 expression (TPS≥1%), but the authors did not analyze the association between PD-L1 expression and clinicopathological variables. Another paper enrolling 17 salivary duct carcinoma patients reported that there was a positive PD-L1 expression rate as high as 53% (13), but in our research, we noted that the rate was 42.4%. This inconsistency might be attributed to several possibilities. First, the antibodies used in immunohistochemistry were different, and a previous study confirmed that the positive rate of PD-L1 expression was significantly affected by the antibody clones (16). Second, the specimens used for PD-L1 detection were different. We used full-face sections, but some used tissue microarray sections. When comparing these two methods, cases with high expression of PD-L1 did not have good concordance (17).

In addition, the term high-grade SGC has been used not only for salivary duct carcinoma but also to refer to some other types of cancer. This was the first study to focus on this small, specific group. We noted that the overall rate of TPS≥1% patients was 43.1%, which was associated with the tumor stage but not the histologic type. This finding was interesting and suggested that the high-grade SGC microenvironment exhibited similar immunogenicity independent of histologic type but was affected by tumor stage. Similarly, Kesar et al. (18) previously noted in their 84 patients that the two most common malignant tumor types presenting with PD-L1 expression were adenocarcinoma not otherwise specified and squamous cell carcinoma.

There are no official standards for reporting PD-L1 expression detection, and three scoring criteria are available: TPS, CPS and the IC score. In the current study, 79.8% and 44.0% of the patients had CPS≥1 and IC scores of 2 or 3, respectively. These incidences were consistent with the reports by Xu et al. (10), Witte et al. (19), and Szewczyk et al. (20). However, there was a discrepancy in the association between PD-L1 expression and clinicopathological variables. Witte et al. (18) noted that both TPS and the IC score were not related to node-positive disease, but a higher CPS means a higher frequency of lymph node metastasis. Possible explanations were differences between the studied cases and the different antibodies used.

The survival effect of PD-L1 expression in SGC is another important issue for analysis. Mukaigawa et al. (7) found that in 219 SGC patients, the 5-year DFS rates of patients showing tumor cell PD-L1 positivity and PD-L1 negativity were 20.2% and 54.6%, respectively, and the difference was significant. The 5-year OS rates of patients with tumor cell PD-L1 positivity and PD-L1 negativity were 40.8% and 80.9%, respectively, and the difference was also significant. In a paper by Xu et al. (10), the authors described that PD-L1 immunopositivity in at least 25% of tumor cells was associated with decreased disease-specific survival. Similar results were also confirmed by Sato et al. (11, 21), Nakano et al. (22), and our results. This suggested a negative survival effect of high TPS. It was the strongest point of current study, we firstly employed the three established predictive scoring criteria on this small and specific group of high grade SGC, and uncovered that TPS rather than CPS or IC score showed better promising target for immuno-oncologic treatment.

However, there were also totally different viewpoints. Higashino et al. (9) showed that in their 127 patients, disease-specific survival was 86.9% for those with PD-L1-negative tumors and 82.2% for patients with PD-L1-positive tumors, and there was no significant difference. In their subgroup analysis of high grade cancers, it was 52.7% in 23 patients with PD-L1-negative tumors and 62.5% in 21 patients with PD-L1-positive tumors, again showing no significant difference, and it remained the same in low- and intermediate grade cases. Even when expression by 10% of tumor cells was used as the threshold for defining PD-L1 positivity, no significant difference in disease-specific survival was observed. Hamza et al. (12) included 113 patients, and the authors reported that the OS rates at 3, 5 and 10 years were 52.6%, 37.9% and 25.6%, respectively. There was no significant difference in survival between patients with PD-L1-immunoreactive tumors and those without. Similar findings were also reported by Schvartsman et al. (13) and Witte et al. (19). Vital et al. (8) reported that PD-L1 expression in tumor cells did not have any correlation with DFS and OS in 167 patients with SGC, but PD-L1 positivity in tumor-infiltrating immune cells predicted a worse DFS and OS in salivary duct carcinoma. Xu et al. (10) noted PD-L1 immunopositivity determined with a cutoff of CPS≥1 was associated with improved disease-specific survival and DFS in salivary duct carcinoma. The presence of PD-1-positive immune cells was associated with improved survival regardless of the expression level. However, in our study, we failed to note a positive relationship between prognosis and CPS/IC score. The differences might be explained by that the authors investigated only PD-L1 positive tumor cells and did not check the PD-1 positive immune cells, and more importantly, even if there were many PD-L1 positive tumor cells, sometimes there were few PD-1 positive immune cells around the tumor cells. Usually, PD-1 positive immune cells induced PD-L1 positive tumor cells as a result of immune response. However, sometimes tumor cells expressed PD-L1 with innate immune response.

Limitations in the current study must be acknowledged. First, our sample size was relatively small. Second, this was a retrospective study, and it had inherent bias. Third, we used paraffin-embedded specimens and not fresh tissue to assess PD-L1 expression, which might affect the accuracy of PD-L1 expression evaluation. Fourth, PD-L1 expression level showed heterogeneity among different PD-L1 antibodies used and different pathologists, and also it showed intra-tumoral heterogeneity, more studies were needed to clarify the PD-L1 expression level in high-grade SGCs.

In summary, the expression of PD-L1 in tumor cells of high-grade SGCs was not uncommon, TPS was associated with tumor stage and prognosis, and a greater TPS indicated worse survival. Both CPS and IC scores had no relationship with clinicopathological variables or prognosis. Patients with high TPS might benefit from immunotherapy.

## Data Availability Statement

The original contributions presented in the study are included in the article/**Supplementary Material**. Further inquiries can be directed to the corresponding author.

## Ethics Statement

The studies involving human participants were reviewed and approved by Henan cancer hospital institutional research committee approved this study, and all participants signed an informed consent agreement. The patients/participants provided their written informed consent to participate in this study.

## Author Contributions

All authors contributed to the article and approved the submitted version.

## Conflict of Interest

The authors declare that the research was conducted in the absence of any commercial or financial relationships that could be construed as a potential conflict of interest.

## Publisher’s Note

All claims expressed in this article are solely those of the authors and do not necessarily represent those of their affiliated organizations, or those of the publisher, the editors and the reviewers. Any product that may be evaluated in this article, or claim that may be made by its manufacturer, is not guaranteed or endorsed by the publisher.
