# Corrigendum: Incidence and Prognostic Significance of PD-L1 Expression in High-Grade Salivary Gland Carcinoma

**DOI:** 10.3389/fonc.2021.836903

**Published:** 2021-12-30

**Authors:** Qigen Fang, Yao Wu, Wei Du, Xu Zhang, Defeng Chen

**Affiliations:** Department of Head Neck and Thyroid, Affiliated Cancer Hospital of Zhengzhou University, Henan Cancer Hospital, Zhengzhou, China

**Keywords:** salivary gland carcinoma, PD-L1, immunotherapy, high-grade salivary gland carcinoma, survival

In the published article, there was an error in affiliation order. Instead of “Department of Head Neck and Thyroid, Henan Cancer Hospital, Affiliated Cancer Hospital of Zhengzhou University, Zhengzhou, China”, it should be “Department of Head Neck and Thyroid, Affiliated Cancer Hospital of Zhengzhou University, Henan Cancer Hospital, Zhengzhou, China”.

The authors apologize for this error and state that this does not change the scientific conclusions of the article in any way. The original article has been updated.

## Publisher’s Note

All claims expressed in this article are solely those of the authors and do not necessarily represent those of their affiliated organizations, or those of the publisher, the editors and the reviewers. Any product that may be evaluated in this article, or claim that may be made by its manufacturer, is not guaranteed or endorsed by the publisher.

