# Supplementary figures and images for: Incidence and Prognostic Significance of PD-L1 Expression in High-Grade Salivary Gland Carcinoma
Source: Front Oncol. 2021 Aug 26;11:701181. doi: 10.3389/fonc.2021.701181 (PMC8427307; doi:10.3389/fonc.2021.701181)

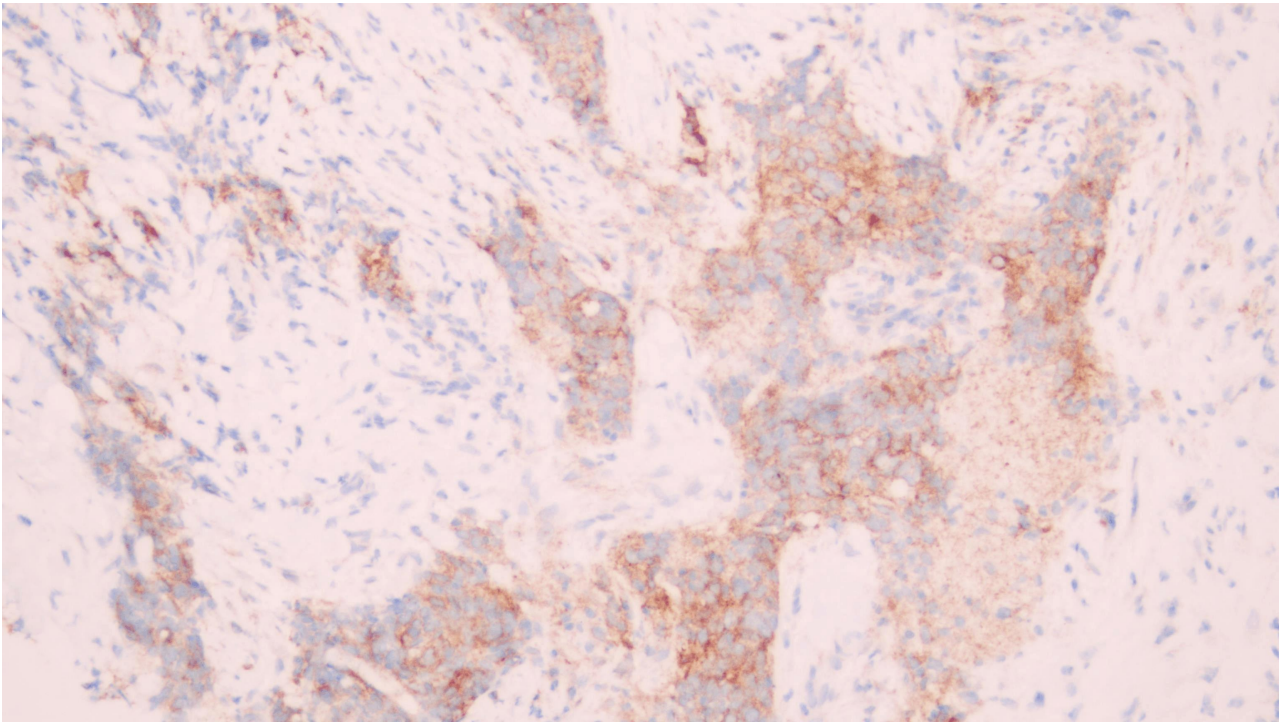

TPS: 8%; CPS: 10; IC: 5% (HE,  $\times 200$ )

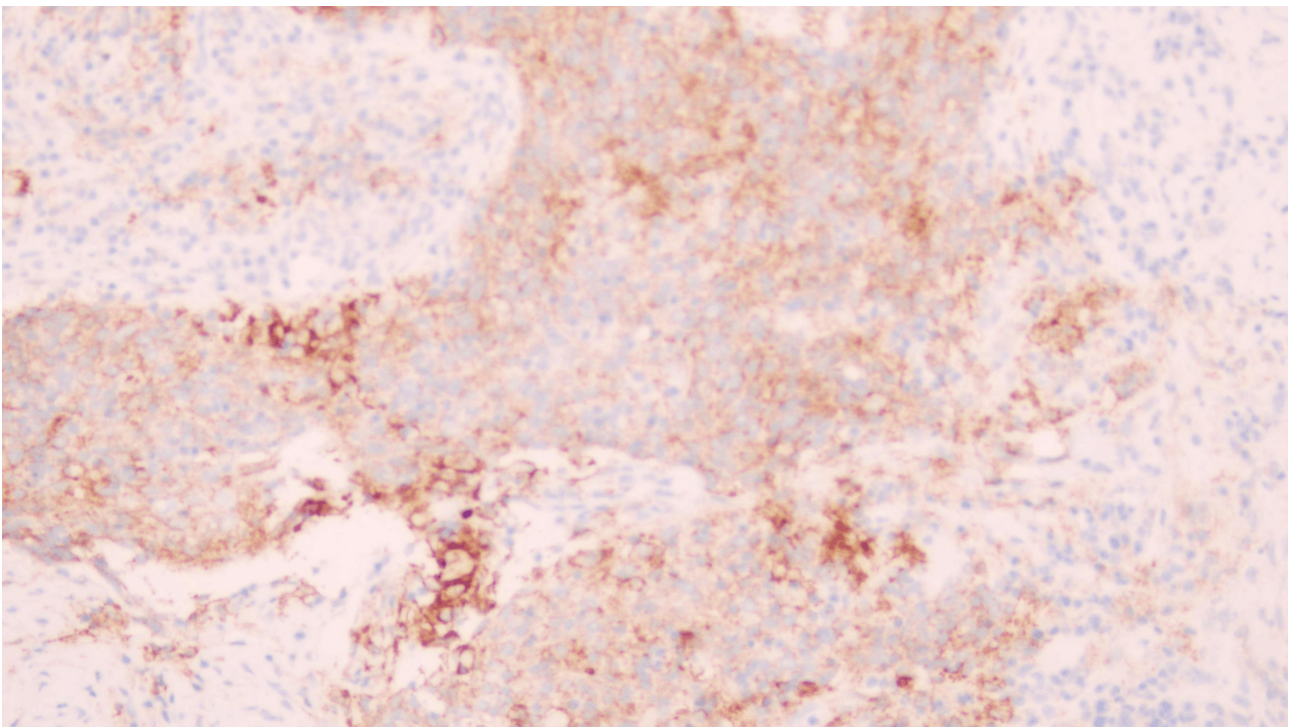

TPS: 30%; CPS: 50; IC: 15% (HE,  $\times 200$ )

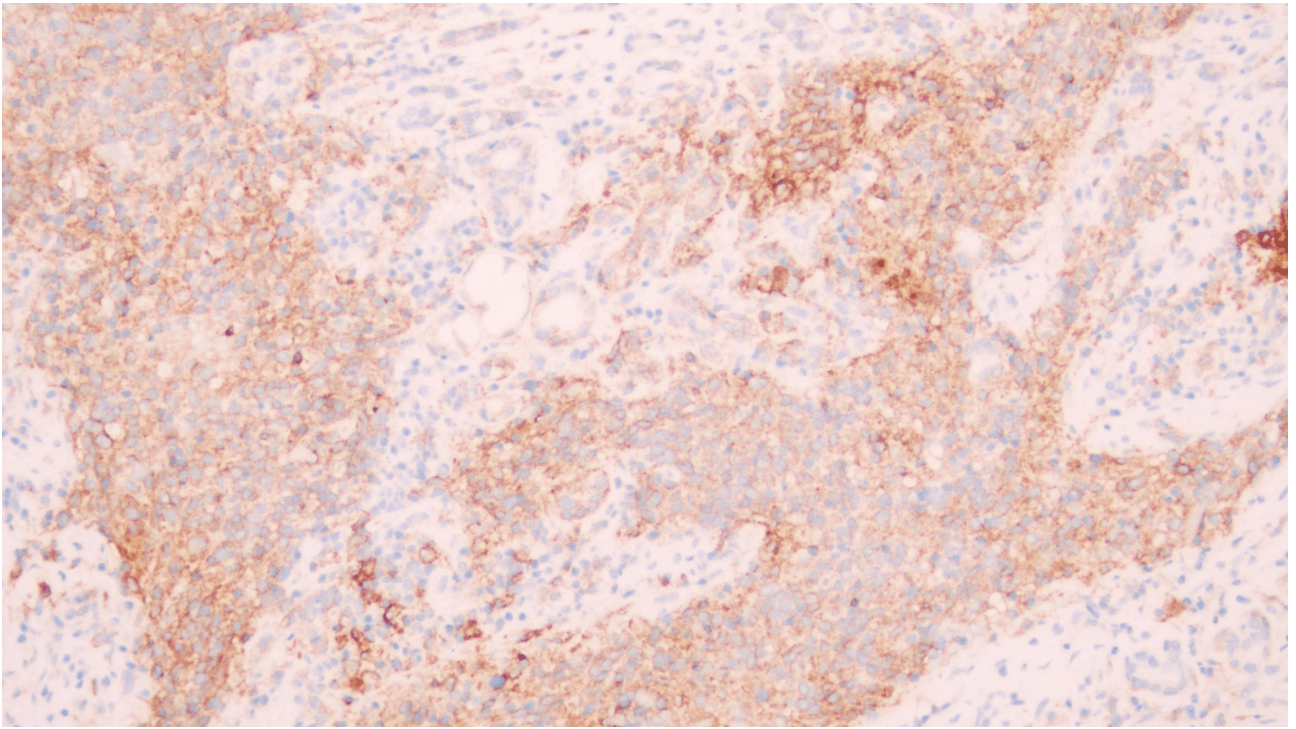

TPS:40%; CPS: 80; IC: 25% (HE,  $\times 200$ )

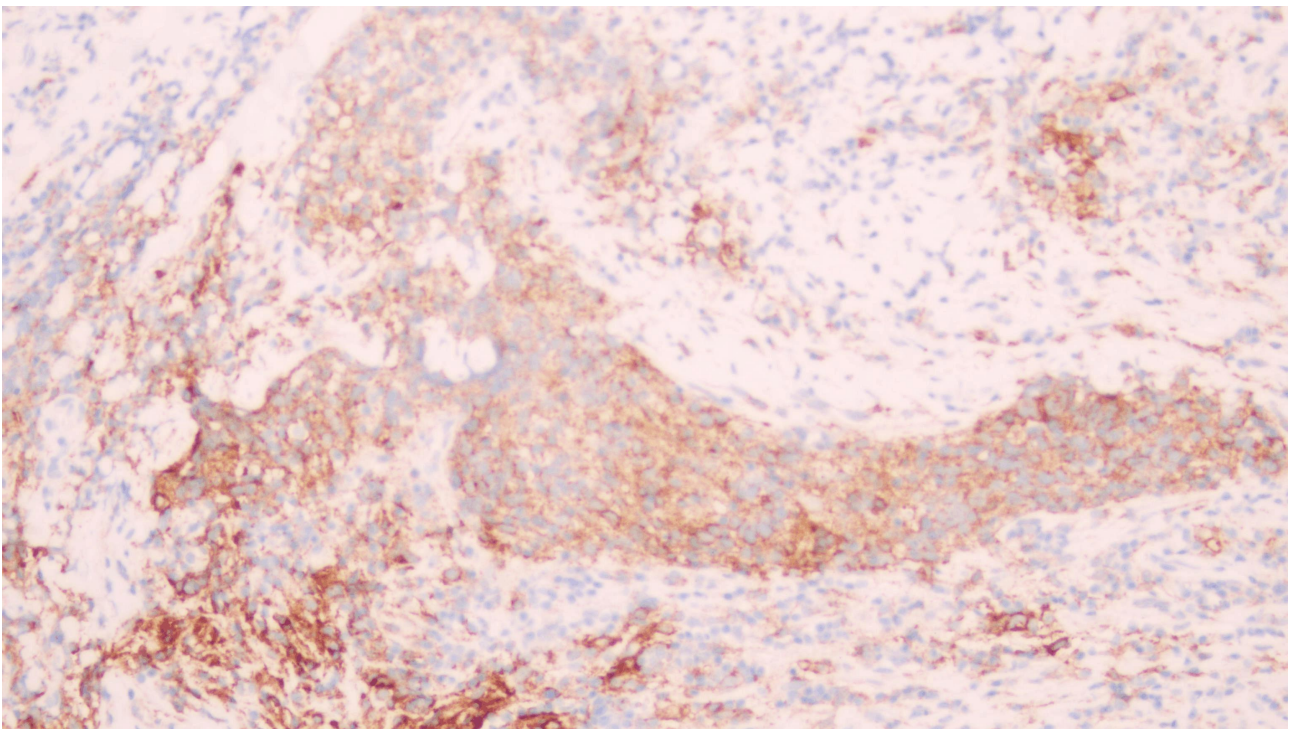

TPS: 28%; CPS: 90; IC: 8% (HE,  $\times 200$ )

Supplement: Supplementary file 1 [file DataSheet_1.pdf]
